# Weight gain following a diagnosis of anti-neutrophil cytoplasmic antibody-associated vasculitis

**DOI:** 10.1093/rap/rkaf088

**Published:** 2025-08-09

**Authors:** Tania Salehi, Thomas French, Tariq E Farrah, Neeraj Dhaun, Robert W Hunter

**Affiliations:** Department of Renal Medicine, Royal Infirmary of Edinburgh, Edinburgh, Scotland; Central and Northern Adelaide Renal and Transplantation Service, Royal Adelaide Hospital, Adelaide, Australia; Department of Renal Medicine, Royal Infirmary of Edinburgh, Edinburgh, Scotland; Department of Renal Medicine, Royal Infirmary of Edinburgh, Edinburgh, Scotland; Edinburgh Kidney Research Group, Centre for Cardiovascular Science, University of Edinburgh, Edinburgh, Scotland; Department of Renal Medicine, Royal Infirmary of Edinburgh, Edinburgh, Scotland; Edinburgh Kidney Research Group, Centre for Cardiovascular Science, University of Edinburgh, Edinburgh, Scotland; Department of Renal Medicine, Royal Infirmary of Edinburgh, Edinburgh, Scotland; Edinburgh Kidney Research Group, Centre for Cardiovascular Science, University of Edinburgh, Edinburgh, Scotland

**Keywords:** anti-neutrophil cytoplasm antibody, vasculitis, metabolic disease, quality of life, comorbidity/multimorbidity

## Abstract

**Objectives:**

The prevalence of obesity in ANCA-associated vasculitis (AAV) has not been well documented, despite an increased risk of cardiometabolic disease in these patients. We aimed to characterize changes in weight following a diagnosis of AAV and determine risk factors for gaining weight and becoming obese.

**Methods:**

We examined data from a single-centre registry of patients with AAV, diagnosed between 2003 and 2023. We evaluated changes in weight and BMI following diagnosis and determined the prevalence of obesity. Using linear regression, we identified factors contributing to an increase in BMI at 6 months. Logistic regression was used to define predictors for obesity at 6 months.

**Results:**

A total of 215 patients with AAV were included. Patients experienced a mean weight gain of 5.2% in the first 6 months; this was sustained for at least 2 years. A total of 69% of patients were overweight or obese at 6 months compared with 59% at baseline. Weight gain was greater following the first presentation compared with relapsing disease. Baseline factors associated with an increase in BMI at 6 months included higher eGFR [β = 0.70 (95% CI 0.36, 1.03), *P* < 0.001] and earlier year of presentation [β = 0.38 (95% CI 0.08, 0.69), *P* = 0.008]. Higher eGFR [adjusted odds ratio (aOR) 1.36 (95% CI 1.08, 2.72), *P* < 0.001] and baseline BMI [aOR 2.57 (95% CI 1.81, 3.64), *P* < 0.001] were associated with an increased likelihood of obesity at 6 months.

**Conclusion:**

Weight gain is common following a diagnosis of active AAV. This is now less pronounced than it was 2 decades ago. Better kidney function and higher baseline BMI are associated with a greater risk of being obese at 6 months. Many patients with AAV are likely to benefit from interventions aimed at achieving a healthy weight.

## Graphical Abstract

**Figure:**
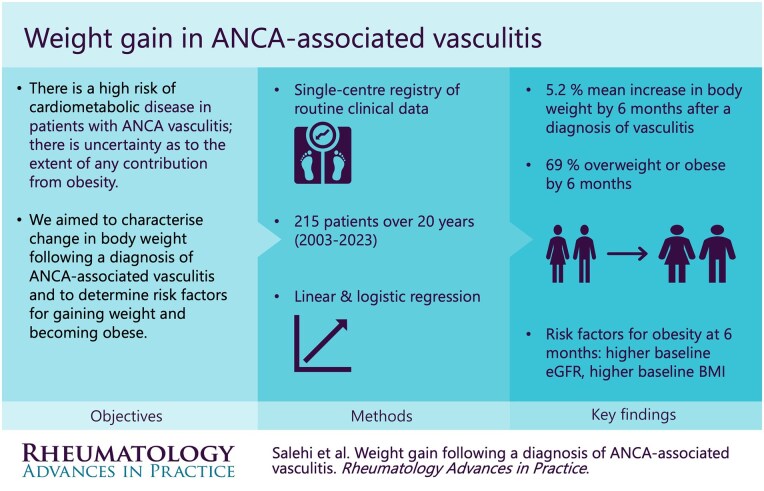

Key messages • Weight gain and an unhealthy high BMI are prevalent following diagnosis of active AAV. • Higher baseline eGFR is associated with greater weight gain in the first 6 months following diagnosis. • Weight gain is less pronounced following treatment of relapsing disease compared with initial presentation.

## Introduction

ANCA-associated vasculitis (AAV) is a rare, potentially life-threatening autoimmune disease characterized by necrotizing inflammation in small vessels, causing end-organ injury [1, 2]. The management of severe AAV involves the use of combination immunosuppression including high-dose glucocorticoids, cyclophosphamide and/or B cell depletion therapy. Short-term mortality has fallen substantially as treatments have improved in recent decades. Thus the major challenges facing individuals with AAV relate to the longer-term sequalae of organ damage and immunosuppressive treatments, including increased rates of cardiometabolic disease [3].

An increase in body weight following a diagnosis of active AAV has been observed in clinical trial populations. Post hoc analyses of the Wegener’s Granulomatosis Etanercept (WGET) and Rituximab in AAV (RAVE) trials demonstrated that AAV patients treated with high-dose glucocorticoid regimens experienced significant weight gain in the first 6–12 months after diagnosis [4, 5]. However, in ‘real-world’ epidemiological studies, patients with AAV do not exhibit increased rates of obesity (whereas they are at increased risk of cardiovascular disease, diabetes mellitus, thyroid disease, osteoporosis and venous thromboembolism) [6–8]. Thus there is uncertainty around the extent of this potential problem outside of clinical trial settings.

Understanding the prevalence of and risk factors for obesity in AAV would allow personalization of treatment regimens to minimize the risk of AAV-associated obesity and its complications. Thus we characterized changes in body weight following a diagnosis of active AAV in a contemporary, real-world cohort and determined the risk factors involved. We aimed to determine both the risk factors for weight gain (to understand why individuals gain weight) and the risk factors for becoming obese (to identify those individuals who could benefit from therapeutic interventions designed to maintain a healthy body weight). In a secondary analysis, we determined whether obesity modifies the risk of disease relapse, given that obesity and metabolic syndrome are known to induce a pro-inflammatory state [9, 10].

## Methods

### Study design and population

We analysed data from a single-centre registry of all patients presenting with active AAV between 2003 and 2023. As this was a retrospective review, it met the criteria for a service evaluation study and thus did not require approval from a research ethics committee. ‘Active’ disease was classified based on a combination of clinical, laboratory, radiologic and histologic findings suggesting vasculitis disease activity, with exclusion of alternative causes, that required immunosuppressive treatment. In addition to PR3- and MPO-positive patients, those with ANCA-negative pauci-immune small vessel vasculitis were included in the analysis. We analysed routinely collected clinical and laboratory data retrieved from the electronic health record using Structured Query Language–based database queries. Our study analysed all data from vasculitis clinic visits as well as any other blood tests taken in primary or secondary care in our health board.

### Patient characteristics

At the time of diagnosis of active AAV we recorded age, sex, weight, BMI, diabetic status, lipid profile, serum creatinine, estimated glomerular filtration rate (eGFR), urine protein:creatinine ratio (UPCR) and ANCA serology. During the treatment and follow-up phases we recorded induction therapeutic agent(s), glucocorticoid exposure and relapse history.

The baseline weight was taken as the measurement closest to the date of AAV diagnosis, accepting any value within a window extending 90 days preceding and 30 days following diagnosis. Weight was recorded at every follow-up visit to the vasculitis clinic. Weights at 6 months and 2 years after presentation were taken as the measurement closest to 180 and 730 days after diagnosis, respectively, accepting any value within a window extending 90 days preceding and 90 days after. Height measurements were presumed to have remained unchanged throughout the study. In the event of multiple, discrepant height recordings for one individual, the median value was taken. BMI was classified into four subcategories corresponding to World Health Organization definitions [11]: underweight, BMI <18.5 kg/m^2^; healthy weight, BMI 18.5–24.9 kg/m^2^; overweight, BMI 25.0–29.9 kg/m^2^; obese, BMI ≥30.0 kg/m^2^.

Baseline diabetes and hyperlipidaemia were inferred from blood test data. A diagnosis of diabetes was assumed if there was a recorded haemoglobin A1c level ≥48 mmol/mol and/or a random blood glucose level ≥11.1 mmol/l at or prior to diagnosis. Patients were deemed to have hypercholesterolaemia in the presence of an elevated low-density lipoprotein cholesterol of >3 mmol/l and/or total cholesterol >5 mmol/l at or prior to diagnosis. eGFR was calculated using the Chronic Kidney Disease Epidemiology Collaboration equation [12]. ANCA status was classified as PR3 positive, MPO positive, dual PR3 and MPO positive or ANCA negative, according to the highest recorded value for the PR3 and MPO ELISA titre at any time during follow-up. Induction immunosuppression was classified as being cyclophosphamide monotherapy, rituximab monotherapy, combination cyclophosphamide and rituximab or mycophenolate mofetil monotherapy. The doses of glucocorticoids used were documented both at the beginning of treatment (week 0) and at week 12 and were reported as prednisolone equivalents.

Remission was defined as clinically silent disease in a patient taking no more than 7.5 mg prednisolone per day. Relapse was defined as a change in symptoms or signs that—after appropriate investigation—was likely to be attributable to vasculitis disease activity and not to an alternative pathology such as infection. In almost all instances this prompted an increase in immunosuppression. All diagnoses of ‘active’ AAV, ‘relapse’ and ‘remission’ were made by a consultant physician with experience managing AAV patients.

### Endpoints

Our primary analysis determined how body weight and BMI change following a diagnosis of active AAV. We evaluated the trajectory of body weight over time post-diagnosis and initiation of immunosuppression for the index AAV presentation and after the first episode of disease relapse. We determined the prevalence of an unhealthy high BMI at diagnosis and during follow-up. We sought to identify baseline risk factors for an increasing BMI and obesity at 6 months. In an exploratory secondary analysis, we examined whether obesity was associated with differences in rates of AAV relapse.

### Statistical analysis

Continuous variables were reported as group means (s.d.) if normally distributed or as median [interquartile range (IQR)] if not.

The average relative differences in baseline characteristics between PR3- and MPO-positive cohorts were compared by two-tailed *t*-tests for normally distributed continuous variables, Mann–Whitney U tests for non-normally distributed continuous variables and chi-squared tests for categorical variables. Paired two-tailed *t*-tests were used to evaluate changes in weight over time.

Ordinary least squares regression was used to identify predictors of percentage change in BMI at 6 months. In order to account for the non-linear relationship between age and change in BMI, a quadratic term was included in regression formulae concerning this variable (age + age^2^). Unadjusted regression coefficients were calculated through univariable linear regression and adjusted regression coefficients were calculated adjusting for age, BMI at baseline, eGFR, induction agent, glucocorticoid dose at week 0 and year of presentation. Controlling variables for each regression methodology were identified via a backwards stepwise approach [13] using the Akaike information criterion as a measure of model comparison. In order to approximate a normal distribution in the dependent variable (BMI percentage change at 6 months), seven outliers (identified by a distance from the median of >1.5 times the IQR) were not included in the final analysis. Assumptions of normality and heteroscedasticity were assessed using a Q-Q plot of residuals and a predicted/observed scatter plot of residuals.

Univariable and multivariable logistic regression were used to calculate odds ratios (ORs) for obesity at 6 months from induction therapy. Adjusted ORs (aORs) were calculated adjusting for age, sex, baseline BMI, eGFR, ANCA subgroup, induction agent, glucocorticoid dose at week 0 and week 12 and year of presentation. The ‘year of presentation’ refers to the year in which the active AAV episode was diagnosed. We adjusted for this variable because treatment strategies have evolved significantly during the study period from 2003 to 2023 and this might have affected changes in body weight.

Time-to-event analysis was performed to identify risk factors for disease relapse. Time to either relapse or censorship from death or loss to follow-up was used to generate survival tables. Cox proportional hazards regression was used to generate hazard ratios adjusted for age, sex, gender, ANCA subgroup and induction agent. Kaplan–Meier curves were compared with a logrank test.

Missing data were treated as missing completely at random and regression analyses were completed on a ‘complete case’ basis. R software version 4.3.2 (R Foundation for Statistical Computing, Vienna, Austria) was used for statistical analysis, along with the packages readxl, plyr, tidyr, pROC, survival, ggsurvfit and car.

## Results

### Baseline characteristics

During the study period a total of 436 patients were diagnosed with the first presentation AAV, of whom 215 had available baseline BMI data and were included in the analysis. The vast majority of those with missing BMI data had no recorded height. Patients were followed up for a median of 5.7 years (IQR 2.4–9.4).

Baseline demographic, clinical and laboratory characteristics of the cohort are presented in Table 1. Baseline features stratified by BMI category are presented in Supplementary Table S1, available at *Rheumatology Advances in Practice* online. The median baseline BMI was 26.0  kg/m^2^ (IQR 22.9–30.1). A total of 70 patients (33%) were overweight and 56 patients (26%) were obese at presentation with AAV. In nine patients, the induction immunosuppression regimen did not fall into our prespecified categories (and was therefore not classified). A total of 52 patients (24.2%) experienced one or more clinical relapses during the follow-up period. Six patients (2.8%) had refractory disease and did not achieve clinical remission.

**Table 1.** Demographic, clinical and laboratory characteristics of AAV patients (*N* = 215) at baseline.^a^

Characteristics	Values
Age (years), median (IQR)	65.5 (51.8–74.4)
Male, *n* (%)	99 (46.0)
Baseline weight (kg), mean (s.d.)	74.4 (17.5)
BMI (kg/m^2^), median (IQR)	26.0 (22.9–30.1)
Creatinine^a^ (μmol/l), median (IQR)	153 (75–290)
eGFR^a^ (ml/min/1.73 m^2^), median (IQR)	34.1 (16.0–79.6)
UPCR^a^ (mg/mmol), mean (s.d.)	86 (128)
Diabetes^a^, *n* (%)	14 (6.5)
Hypercholesterolaemia^a^, *n* (%)	78 (36.3)
ANCA status, *n* (%)	
PR3	79 (36.7)
MPO	76 (35.4)
Dual positive	34 (15.8)
Negative	26 (12.1)
Induction therapy^a^, *n* (%)	
Cyclophosphamide	67 (31.2)
Rituximab	48 (22.3)
Rituximab + cyclophosphamide	51 (23.7)
MMF	40 (18.6)
Prednisolone dose (mg), median (IQR)	
Week 0^a^	60 (40–60)
Week 12^a^	10 (5–12.5)
Relapse history, *n* (%)	
No relapse episodes	157 (73.0)
One or more relapse episodes	52 (24.2)
Non-remitting^b^	6 (2.8)

a Number of missing values: creatinine = 3, eGFR = 3, UPCR = 91, diabetes = 90, hypercholesterolaemia = 108, induction therapy = 9, prednisolone dose week 0 = 7, prednisolone dose week 12 = 9.

b Remission not achieved following induction therapy.

Baseline characteristics comparing PR3- and MPO-positive cohorts are shown in Table 2. PR3-AAV patients had a significantly higher baseline weight and BMI compared with MPO-AAV patients. PR3-AAV patients had better renal function at the time of diagnosis and were more likely to have received cyclophosphamide induction therapy.

**Table 2.** Comparison of baseline characteristics between PR3- and MPO-AAV.

Characteristics	PR3 ANCA (*n* = 79)	MPO ANCA (*n* = 76)	*P*-value
Age (years), mean (s.d.)	57.4 (16.8)	68.2 (14.4)	0.18
Male, *n* (%)	38 (48.1)	38 (50.0)	0.81
Baseline weight (kg), mean (s.d.)	78.0 (20.9)	70.4 (15.6)	0.01
BMI (kg/m^2^), mean (s.d.)	27.3 (6.3)	24.9 (5.0)	0.045
Creatinine^a^ (μmol/l), median (IQR)	127 (71–234)	196 (111.5–322.8)	0.02
eGFR^a^ (ml/min/1.73 m^2^), median (IQR)	46.2 (21.2–95.5)	26.1 (13.9–50.9)	0.003
UPCR^a^ (mg/mmol), mean (s.d.)	72 (102)	94 (137)	0.20
Diabetes^a^, *n* (%)	2 (2.5)	6 (7.9)	0.13
Hypercholesterolaemia^a^, *n* (%)	27 (34.2)	28 (36.8)	0.73
Induction therapy, *n* (%)			
Cyclophosphamide	33 (41.8)	19 (25.0)	0.03
Rituximab	16 (20.3)	22 (28.9)	0.21
Rituximab + cyclophosphamide	18 (22.8)	23 (30.3)	0.29
MMF	10 (12.7)	11 (14.5)	0.74
Prednisolone dose (mg), median (IQR)			
Week 0^a^	60 (40–60)	60 (40–60)	0.76
Week 12^a^	10 (5–12.5)	10 (5–12.5)	0.09

PR3 ANCA subgroup: diabetes = 38, hypercholesterolaemia = 48, prednisolone dose week 0 = 4, prednisolone dose week 12 = 4.

MPO ANCA subgroup: creatinine = 2, eGFR = 2, diabetes = 29, hypercholesterolaemia = 34.

a Number of missing values.

### Body weight trajectory after AAV diagnosis or relapse

**Figure 1.**
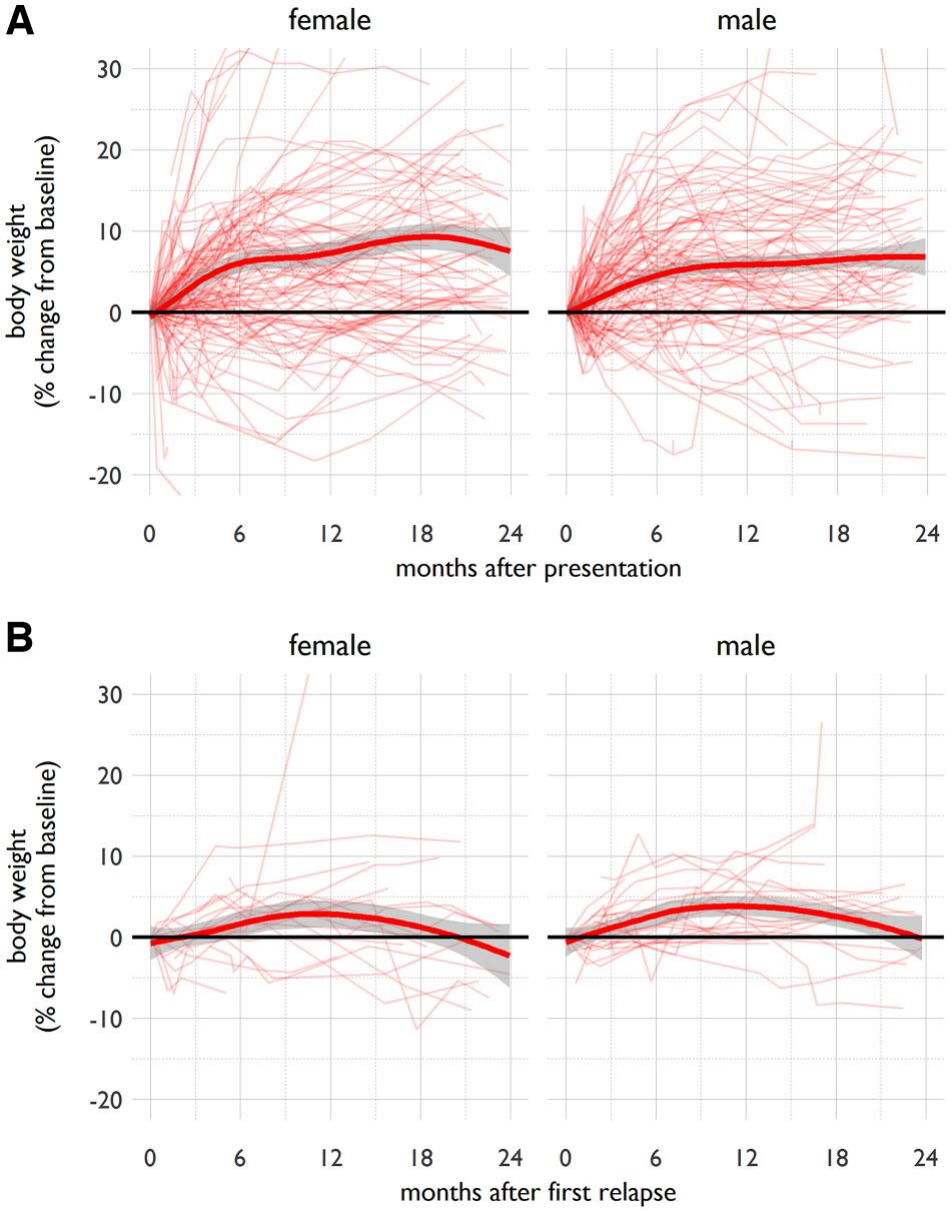
Trends in body weight following **(A)** first presentation with AAV and **(B)** first episode of relapse of AAV. Body weight for individuals plotted relative to body weight at the time of presentation with vasculitis or first episode of disease relapse. The heavy line is the population average (smoothed conditional mean computed using a generalized additive model)

Most patients (62.8%) gained weight during the first 6 months following AAV diagnosis and sustained this weight gain for at least 2 years (Fig. 1A). On average, patients gained a mean 5.2% (s.d. 8.2%, *P* = 0.001) of body weight by 6 months. During this period, a BMI increase ≥5% was experienced by 87 patients (47.3%) and the median BMI increased to 27.5 kg/m^2^ (IQR 23.6–31.4) [+1.5 kg/m^2^ (IQR 0.7–1.3)] with 66 patients (35%) documented as being overweight and 63 (34%) obese. Given this trajectory, we selected percentage change in BMI at 6 months as an appropriate endpoint in the linear regression analyses presented below, particularly due to the acknowledged and clinically meaningful effects of weight fluctuations falling within the 3–5% range on overall well-being and cardiovascular risk [14, 15]. The changes in individuals’ BMI categories from baseline to 6 months are shown in Supplementary Fig. S1 and Table S2, available at *Rheumatology Advances in Practice* online.

Weight gain following the first episode of disease relapse showed a less pronounced trend and reached its peak later after treatment (Fig. 1B). Lower glucocorticoid doses were administered during disease relapse compared with index presentation treatment with median prednisolone doses of 40 mg (IQR 20–40) and 7.5 mg (IQR 5–10) given at weeks 0 and 12, respectively.

### Risk factors for weight gain

**Figure 2.**
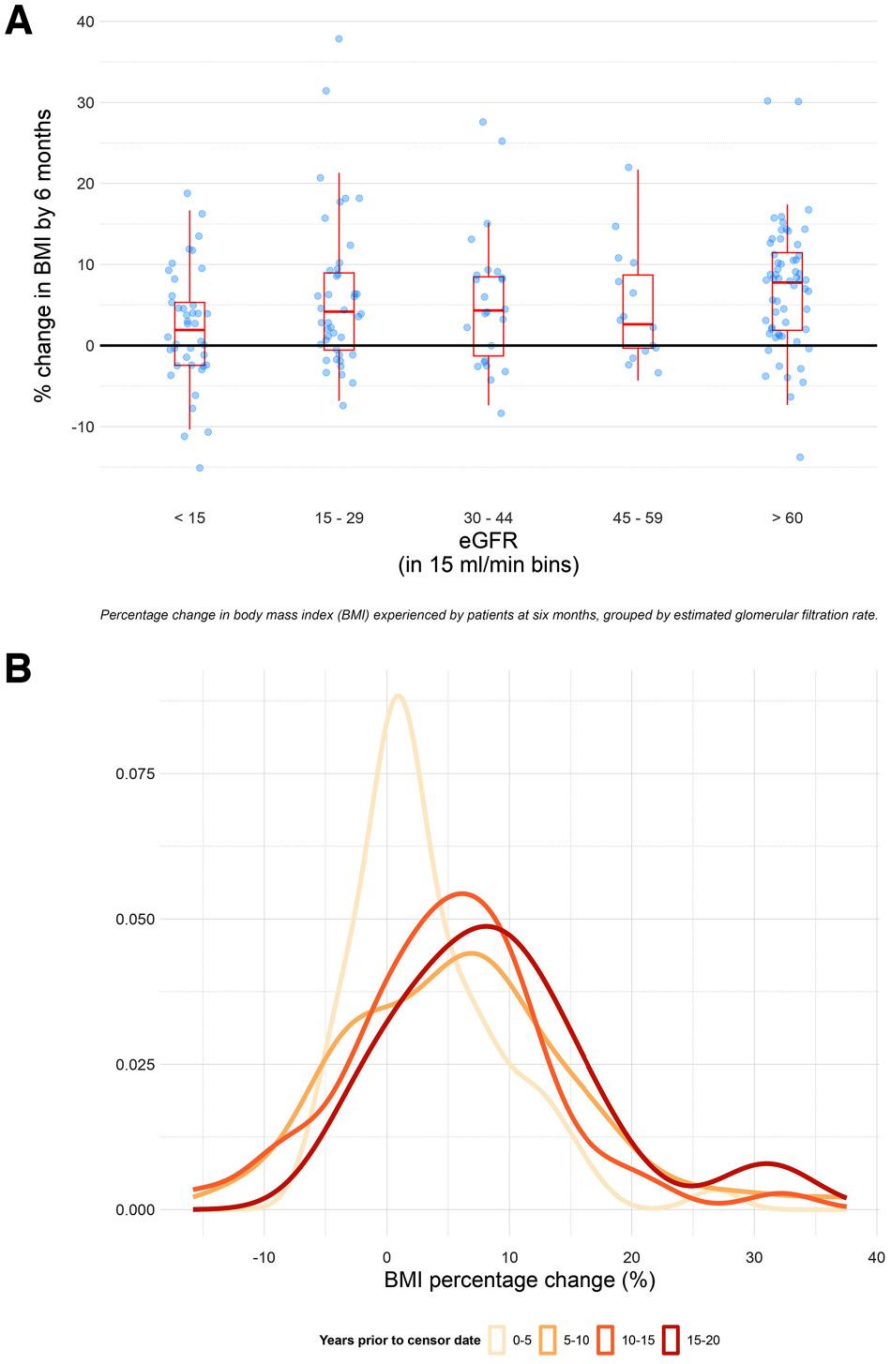
Percentage change in BMI experienced by patients at 6 months, grouped by **(A)** eGFR and **(B)** year of presentation to the vasculitis service

Baseline characteristics associated with the change in BMI at 6 months were a higher baseline eGFR [β = 0.70 (95% CI 0.36, 1.03), *P* < 0.001] and an earlier year of presentation [β = 0.38 (95% CI 0.08, 0.69), *P* = 0.008] (Table 3). A 10 ml/min/1.73 m^2^ increase in eGFR corresponded to a BMI increase of 0.70% at 6 months from baseline. Those with an eGFR >60 ml/min/1.73 m^2^ experienced the greatest increase in BMI, with significant interindividual variability (Fig. 2A). Additionally, for each year that the presentation date was in advance of the study’s conclusion there was a corresponding 0.38% increase in BMI (Figures 2B). The *R*^2^ value for the adjusted model was 0.102, meaning that 10.2% of the observed variation in BMI percentage change was explained by the linear regression model.

**Table 3.** Determinants of BMI percentage change at 6 months.

Characteristics	Unadjusted regression coefficients			Adjusted^a^ regression coefficients		
	β	95% CI	*P*-value	β	95% CI	*P*-value
Year of presentation	0.22	0.01, 0.44	0.039	0.38	0.08, 0.69	0.008
Age (years)	0.34	0.19, 1.74	0.084	0.29	−0.15, 0.73	0.35
Age^2^ (years^2^)	−0.05	−0.01, 0.00	0.077	−0.01	−0.01, 0.00	0.52
Sex						
Male	0 (ref)			0 (ref)		
Female	1.15	−0.83, 3.13	0.253	0.77	−1.27, 2.77	0.45
BMI at baseline	−0.10	−0.26, 0.07	0.245	−0.17	−0.35, 0.01	0.06
eGFR (10 ml/min/1.73 m^2^)	0.37	0.09, 0.64	0.009	0.70	0.36, 1.03	<0.001
UPCR	−0.002	−0.01, 0.00	0.380	0.00	−0.01, 0.01	0.58
Diabetes	−1.28	−5.08, 2.53	0.508	−0.72	−5.05, 3.62	0.74
Hyperlipidaemia	−1.40	−4.58, 1.79	0.385	−2.53	−5.83, 0.78	0.13
ANCA subgroup						
Negative	0 (ref)			0 (ref)		
MPO	−0.10	−2.16, 1.96	0.925	0.52	−1.69, 2.73	0.64
PR3	1.22	−0.81, 3.25	0.238	0.75	−1.52, 3.01	0.52
Dual positive	−0.75	−3.51, 2.01	0.594	−0.91	−3.80, 1.97	0.53
Induction agent						
Cyclophosphamide	1.68	−0.42, 3.78	0.116	0.83	−2.51, 4.17	0.63
Rituximab	0 (ref)			0 (ref)		
Rituximab + cyclophosphamide	−0.68	−3.02, 1.66	0.565	0.04	−0.71, 5.58	0.13
MMF	0.07	−2.37, 2.53	0.951	2.44	−3.22, 3.30	0.98
Prednisolone dose						
Week 0	0.04	−0.01, 0.10	0.105	0.05	−0.01, 0.10	0.12
Week 12	0.03	−0.13, 0.19	0.683	−0.03	−0.23, 0.16	0.74

Adjusted *R*^2^ for final model: 10.2%.

a Adjusted for age, BMI at baseline, eGFR, induction regime and prednisolone dose at week 0 (i.e. the initial prednisolone dose when induction therapy was commenced).

A higher baseline eGFR [aOR 1.36 (95% CI 1.08, 2.72), *P* < 0.001] and higher baseline BMI [aOR 2.57 (95% CI 1.81, 3.64), *P* < 0.001] conferred a significantly increased likelihood of obesity at 6 months from diagnosis (Table 4). The area under the receiver operating characteristics curve for the adjusted regression model decreased from 0.97 to 0.70 when baseline BMI was not included in the logistic regression model, demonstrating that baseline BMI accounts for much of the predictive power of this model.

**Table 4.** ORs for obesity at 6 months from baseline.

Characteristics	OR	95% CI	*P*-value	aOR^a^	95% CI	*P*-value
Year of presentation	1.02	0.96, 1.09	0.52	1.09	0.88, 1.34	0.43
Age (deciles)	0.79	0.65, 0.97	0.02	1.66	0.94, 2.93	0.08
Sex						
Male	1.00 (ref)	NA	NA	1.00 (ref)	NA	NA
Female	0.84	0.45, 1.55	0.57	0.92	0.23, 3.70	0.91
BMI at baseline	2.08	1.66, 2.60	<0.001	2.57	1.81, 3.64	<0.001
eGFR (10 ml/min/1.73 m^2^)	1.09	1.00, 1.19	0.04	1.36	1.08, 2.72	<0.001
UPCR	1	0.99, 1.00	0.95	1.00	0.99, 1.00	0.51
Diabetes	1.32	0.37, 4.77	0.67	1.11	0.25, 4.99	0.89
Hyperlipidaemia	0.48	0.18, 1.28	0.14	0.45	0.11, 1.86	0.27
ANCA subgroup						
Negative	1.00 (ref)	NA	NA	1.00 (ref)	NA	NA
MPO	0.46	0.23, 0.90	0.02	0.33	0.02, 4.92	0.42
PR3	2.09	1.12, 3.93	0.02	1.11	0.11, 10.9	0.93
Dual positive	1.05	0.46, 2.43	0.9	0.26	0.02, 3.40	0.30
Induction agent						
Cyclophosphamide	0.84	0.43, 1.62	0.6	0.4	0.06, 2.89	0.36
Rituximab	1.00 (ref)	NA	NA	1.00 (ref)	NA	NA
Rituximab + cyclophosphamide	0.58	0.30, 1.25	0.16	0.84	0.09, 8.08	0.88
MMF	1.68	0.80, 3.51	0.17	0.25	0.03, 2.41	0.23
Prednisolone dose						
Week 0	1.02	1.00, 1.04	0.02	0.99	0.94, 1.04	0.67
Week 12	1.03	0.98, 1.08	0.27	1.01	0.88, 1.17	0.89

a Adjusted for age, gender, baseline BMI, eGFR, induction agent, prednisolone dose at baseline and 12 weeks and ANCA subgroup.

Baseline BMI was not strongly associated with weight gain after a diagnosis of AAV (Supplementary Figs S2 and S3, available at *Rheumatology Advances in Practice* online).

### Association between obesity and relapse rate

In order to test for an association between obesity and relapse rate, we performed a multivariable Cox proportional hazards regression (Supplementary Table S3, available at *Rheumatology Advances in Practice* online). A baseline BMI ≥30 kg/m^2^ was not associated with an increased risk of relapse after adjusting for confounding variables (HR *P* = 0.85) (Supplementary Fig. S4, available at *Rheumatology Advances in Practice* online). The cumulative relapse hazard curves of obese and non-obese patients did overlap (logrank *P* < 0.001).

## Discussion

### Individuals gain weight after a diagnosis of AAV

In a real-world setting, we showed that individuals tend to experience large gains in body weight after a diagnosis of AAV. Similar to a clinical trial population [4], we found that weight gain typically occurs within the first 6 months after diagnosis. By 6 months, median BMI had increased by 1.5 kg/m^2^, nearly half of the patients had experienced a ≥5% increase in BMI and more than two-thirds of patients were either overweight or obese (compared with ≈59% of patients at baseline). Importantly, we found that changes in body weight and BMI were sustained for years. This is expected to have significant deleterious health consequences. A 5% gain in weight is associated with clinically meaningful increases in the risks of cardiovascular and metabolic diseases [14, 15]. It is also associated with reductions in quality of life [16–18].

We found that the extent of weight gain observed following the first episode of disease relapse was less pronounced, echoing findings from the RAVE trial [4]. Our steroid-prescribing data suggest that this likely reflects reduced glucocorticoid exposure during treatment of relapse (*vs* the first presentation). It is possible that this is also explained by a shorter diagnostic delay, limiting the duration of any hypercatabolic state causing weight loss prior to treatment initiation.

### Risk factors for weight gain

In our cohort, the baseline factors associated with an increase in BMI included better eGFR and disease presentation in an earlier calendar year. A higher baseline eGFR was associated with increased odds of being obese at 6 months. The magnitude of this effect was modest: each 10 ml/min/1.73 m^2^ increment in eGFR conferred an ≈36% increased odds of obesity. In our relatively small, single-centre study, it is possible that this association arose through chance or from residual confounding. However, there are plausible explanations for this observation: for example, it is possible that low eGFR might protect against weight gain because of the anorexic effects of uraemia. It would be interesting to see if this observation is replicated in other cohorts. The small *R*^2^ in our analyses supports the presence of other confounding factors not adjusted by selected variables in our regression models.

We did not find a significant association between glucocorticoid exposure and elevated BMI. This should not be interpreted as evidence that glucocorticoids are not the predominant drivers of weight gain. Indeed, it is overwhelmingly likely that the weight gain experienced after AAV diagnosis is predominantly driven by glucocorticoid therapy, especially given the observed improvement in degree of weight gain in more contemporary treatment eras within our cohort. The lack of any association likely reflects a lack of variation in glucocorticoid dose at the observed time points. Our observation that calendar year of presentation was associated with BMI changes, with greater weight gain for patients presenting longer ago, is likely explained by a trend towards more conservative steroid prescribing over the 2 decades spanned by our study. The changes in our centre’s immunosuppression therapy over time, specifically induction regimens and glucocorticoid dosages, are reflected in Supplementary Figs S5 and S6, available at *Rheumatology Advances in Practice* online.

Unsurprisingly, high BMI at presentation was a strong risk factor for high BMI at 6 months. Therefore, baseline BMI may be a useful parameter to include when prioritizing individuals for steroid-sparing therapies. ANCA serotype was not associated with a differential rate of weight gain. However, our observations align with previous published literature, indicating that PR3-AAV patients present with higher baseline body weight and BMI compared with MPO-AAV [10]. In keeping with the literature [19, 20], MPO-positive patients in our cohort exhibited inferior renal function at the time of diagnosis. The lower baseline BMI we observed within this subgroup could potentially be explained by the often-delayed diagnosis of MPO-AAV resulting in a more prolonged period of constitutional symptoms and uraemia.

### Association between obesity and relapse risk

We did not detect an association between obesity and risk of AAV disease relapse. This is noteworthy because it has been hypothesized that the relapsing nature of PR3-AAV might be exacerbated by the pro-inflammatory state induced by baseline obesity [10, 19].

### Strengths and limitations

We provide the most comprehensive characterization to date of changes in body weight and BMI following a diagnosis of AAV in a contemporary ‘real-world’ cohort.

There are several limitations, in addition to the general limitations inherent in an observational study. We were unable to include the full cohort of AAV patients diagnosed during the study period (*n* = 436), due to the lack of baseline BMI data in 221 patients. Our population is predominantly White Caucasian, limiting the generalizability of our findings. We were unable to quantify the contribution to weight gain from glucocorticoids because of a lack of variation in glucocorticoid dosing. More than half of the cohort (*n* = 119) received 60 mg of prednisolone at treatment onset and only one-third (*n* = 67) had their dose tapered to 10 mg at 3 months, potentially resulting in a model that underestimated the differential effect of glucocorticoids on weight gain. This question should be addressed by future large-scale prospective studies in an era of lower—and more variable—glucocorticoid use or in randomized controlled trials.

## Conclusion and perspective

Our study corroborates that weight gain and obesity are common problems for patients with AAV. The substantial majority of patients in our cohort were overweight or obese by 6 months after diagnosis. We found that major risk factors for weight gain were higher baseline renal function and earlier eras of disease diagnosis (with presumed greater cumulative glucocorticoid exposure).

These factors—combined with baseline BMI—could be used to identify patients at risk of becoming overweight, prioritizing individuals for steroid-sparing treatment regimens and pro-active weight management intervention. Any effective intervention would almost certainly be multimodal, including lifestyle changes, conventional cardiovascular risk reduction strategies [21] and possibly novel pharmacological agents promoting weight loss and cardiovascular protection such as sodium–glucose co-transporter 2 inhibitors and glucagon-like peptide-1 agonists [22, 23]. The data we present here are unlikely to change management strategies on their own, but may be helpful in informing future observational and interventional studies.

The crude prevalence of obesity in our study cohort did not differ substantially from the general population. The mean BMI in Scottish adults is 28 kg/m^2^ and 67% of Scottish adults are overweight or obese, statistics that are replicated almost exactly in our cohort of patients at 6 months after AAV diagnosis [24]. Two years after a diagnosis of AAV, 77% of patients in our cohort were overweight or obese, but these data should be treated with some caution given the relatively low number of patients with BMI data at this time point (*n* = 121). Even if individuals with AAV were not at increased risk of becoming obese, many may still wish to prioritize efforts to achieve and maintain a healthy body weight. AAV is associated with an increased risk of cardiovascular disease, hypertension and diabetes [8]. Obesity is an important modifiable risk factor for these morbidities and therefore individuals with AAV may benefit disproportionately from interventions designed to help maintain a healthy body weight.

## Supplementary Material

rkaf088_Supplementary_Data

## Data Availability

The data that support the findings of this study are available from the corresponding author upon request and subject to our being able to satisfy data-sharing agreements that preserve patient confidentiality.
