# Correction to: Weight gain following a diagnosis of anti-neutrophil cytoplasmic antibody-associated vasculitis

**DOI:** 10.1093/rap/rkaf113

**Published:** 2025-11-13

This is a correction to: Tania Salehi, Thomas French, Tariq E Farrah, Neeraj Dhaun, Robert W Hunter, Weight gain following a diagnosis of anti-neutrophil cytoplasmic antibody-associated vasculitis, *Rheumatology Advances in Practice*, Volume 9, Issue 3, 2025, rkaf088, https://doi.org/10.1093/rap/rkaf088.

The originally published version of this manuscript erroneously omitted the name of author Neeraj Dhaun. The publisher apologises for this error, which has been corrected.

