# Supplementary material for: Weight gain following a diagnosis of anti-neutrophil cytoplasmic antibody-associated vasculitis
Source: Rheumatol Adv Pract. 2025 Aug 9;9(3):rkaf088. doi: 10.1093/rap/rkaf088 (PMC12342786; doi:10.1093/rap/rkaf088)

**Supplementary Appendix**

The code used for analysis of the presented data is publicly available in the following repository: https://github.com/TWFrench/ANCA_vasculitis/tree/main.

The baseline demographic, clinical and laboratory characteristics of the cohort are presented according to BMI categories in Supplemental **Table S1**.

**Figure S1** illustrates the changes in BMI categories among individuals from baseline to six months following diagnosis of AAV. **Table S2** displays BMI category distribution at baseline, six months and two years. **Figures S2** and **S3** further demonstrate changes in BMI at six months based on baseline BMI and BMI category.

The results of the multivariate cox proportional hazard regression are displayed in **Table S3**. A baseline BMI of ≥30 kg/m^2^ was not associated with an increased risk of relapse after adjusting for confounding variables (HR 1.07 [0.53-2.16], P=0.846). Kaplan-Meier curves comparing risk of relapse in the obese cohort and the not-obese cohort did not overlap (log-rank P<0.001) (**Figure S4**). Increasing age (HR 1.57 [1.18-2.09], P=0.002), higher baseline eGFR (HR 1.19 [1.08-1.31], P<0.001) and use of cyclophosphamide rather than rituximab (HR 4.43 [1.53-12.8], P=0.006), conferred a greater risk of experiencing one or more relapse episodes in the follow-up period. Rates of relapse in the population treated with cyclophosphamide alone were significantly higher than those in the population treated with any one of the three alternative treatment regimens (log-rank P<0.001). The changes in our centre’s immunosuppression therapy over time, specifically induction regimens and glucocorticoid dosages, are reflected in **Figures S5** and **S6**. As depicted, cyclophosphamide monotherapy was predominantly utilised in earlier time periods in our study. However, we found that the risk of relapse following cyclophosphamide induction was irrespective of the year of presentation and less likely to be confounded by evolutions in glucocorticoid dosing regimens over time. **Figure S6** highlights prednisolone doses at presentation and three months and suggests a more rapid glucocorticoid tapering in recent years.

| **Table S1. Baseline** **Characteristics of Patients according to BMI Category.** | | | | |
| --- | --- | --- | --- | --- |
|  | **Underweight**  (N = 6) | **Healthy**  (N= 85) | **Overweight**  (N=68) | **Obese**  (N=56) |
| **Characteristic** |  |  |  |  |
| Age | 66.1 ± 25.6 | 66.3 ± 17.7 | 63.7 ± 13 | 56.7 ± 13.6 |
| Male | 1 (16.7%) | 37 (43.5%) | 38 (55.9%) | 23 (41.1%) |
| Weight | 48.1 ± 8.7 | 61.9 ± 8.8 | 76.8 ± 9.5 | 93.2 ± 16.7 |
| BMI | 16.9 ± 0.5 | 22.3 ± 1.8 | 27.1 ± 1.4 | 34.7 ± 4.9 |
| eGFR | 64.8 ± 54.3 | 43.4 ± 35.8 | 45.8 ±33.1 | 50.7 ± 36.7 |
| uPCR | 59 ± 71 | 145.8 ± 167.9 | 137.3 ± 138.5 | 135.3 ± 255.2 |
| Diabetes | 0 (0%) | 7 (8.2%) | 3 (4.4%) | 4 (7.1%) |
| Hypercholesterolaemia | 1 (16.7%) | 33 (38.8%) | 25 (36.8%) | 19 (33.9%) |
| ANCA status |  |  |  |  |
| PR3 | 3 (50%) | 26 (30.6%) | 26 (38.2%) | 24 (42.9%) |
| MPO | 2 (33.3%) | 40 (47.1%) | 21 (30.9%) | 13 (23.2%) |
| Dual positive | 1 (16.7%) | 8 (9.4%) | 15 (22.1%) | 10 (17.9%) |
| Negative | 0 (0%) | 11 (12.9%) | 6 (8.8%) | 9 (16.1%) |
| Induction therapy |  |  |  |  |
| Cyclophosphamide | 2 (33.3%) | 24 (28.2%) | 26 (38.2%) | 15 (26.8%) |
| Rituximab | 2 (33.3%) | 19 (22.4%) | 13 (19.1%) | 14 (25.0%) |
| Rituximab+Cylophosphamide | 1 (16.7%) | 28 (32.9%) | 13 (19.1%) | 9 (16.1%) |
| MMF | 0 (0%) | 11 (12.9%) | 15 (22.1%) | 14 (25%) |
| Prednisolone dose – mg |  |  |  |  |
| Week 0 | 45.8 ± 16.3 | 44.8 ± 18.8 | 54.1 ± 16.4 | 58.8 ± 16.0 |
| Week 12 | 15 ± 14.6 | 7.9 ± 5.7 | 9.6 ± 5.6 | 10.6 ± 6.4 |
| Relapse history |  |  |  |  |
| No relapse episodes | 6 (100%) | 71 (83.5%) | 44 (64.7%) | 42 (75.0%) |
| ≥1 relapse episodes | 0 (0%) | 13 (15.3%) | 24 (35.3%) | 14 (25.0%) |

*Plus–minus values are means ± SD.

Abbreviations: AAV = ANCA associated vasculitis; ANCA = Antineutrophil Cytoplasmic Antibody; BMI = Body Mass Index; eGFR = estimated glomerular filtration rate; MMF = Mycophenolate mofetil; MPO = myeloperoxidase; PR3 = proteinase 3; uPCR = urine protein:creatinine ratio.

| **Table S2. BMI Category Distribution Over Time.** | | |
| --- | --- | --- |
| **Timepoint** | **BMI Category** | **n (%)** |
| **Baseline** (N=215) | Underweight | 6 (2.8) |
|  | Healthy | 83 (38.6) |
|  | Overweight | 70 (32.6) |
|  | Obese | 56 (26.0) |
| **6 months** (N = 188) | Underweight | 2 (1.1) |
|  | Healthy | 57 (30.3) |
|  | Overweight | 66 (35.1) |
|  | Obese | 63 (33.5) |
| **2 years** (N = 121) | Healthy | 28 (23.1) |
|  | Overweight | 40 (33.1) |
|  | Obese | 53 (43.8) |

Abbreviations: AAV = ANCA associated vasculitis; BMI = Body Mass Index.

| Table S3. Hazard Ratios for Likelihood of Experiencing ≥1 Relapse Episodes. | | | |
| --- | --- | --- | --- |
| Characteristic | **Adjusted HR** | **95% CI** | **P-value** |
| Baseline obesity | 1.07 | 0.53 -2.17 | 0.846 |
| Presentation year | 0.99 | 1.18-2.09 | 0.867 |
| Age (deciles) | 1.57 | 1.08-1.31 | 0.002 |
| Female sex | 0.98 | 0.55-1.75 | 0.94 |
| eGFR (10 mL/min) | 1.19 | 0.90-1.09 | <0.001 |
| ANCA sub-group^*^ |  |  |  |
| PR3 | 3.08 | 0.89-10.6 | 0.075 |
| MPO | 0.62 | 0.15-2.51 | 0.503 |
| Dual positive | 1.07 | 0.26-4.43 | 0.923 |
| Induction agent^**^ |  |  |  |
| Cyclophosphamide | 4.43 | 1.53-12.8 | 0.006 |
| Rituximab +Cyclophosphamide | 0.28 | 0.03-2.42 | 0.247 |
| MMF | 2.43 | 0.77-7.80 | 0.136 |

^*^Relative to ANCA negative
^**^Relative to induction with rituximab

Abbreviations: ANCA = Anti-neutrophil Cytoplasm Antibody; CI = Confidence interval; eGFR = Estimated glomerular filtration rate; HR = Hazard ratio; MMF = Mycophenolate mofetil; MPO = Myeloperoxidase; PR3 = Proteinase 3; uPCR = Urine protein:creatinine ratio.

**Figure S1.** Changes in BMI category from baseline to six months following diagnosis of AAV. **A) All patients in the study. B) Excluding patients with no available BMI at 6 months.** There were 185 individuals with BMI data at baseline and at 6 months. There were 6 individuals who had died within 6 months of presentation. There were 24 individuals who were still alive at 6 months, but for whom we lacked BMI data because we had not measured a weight within the relevant window (180 days +/- 90 days after presentation).

**
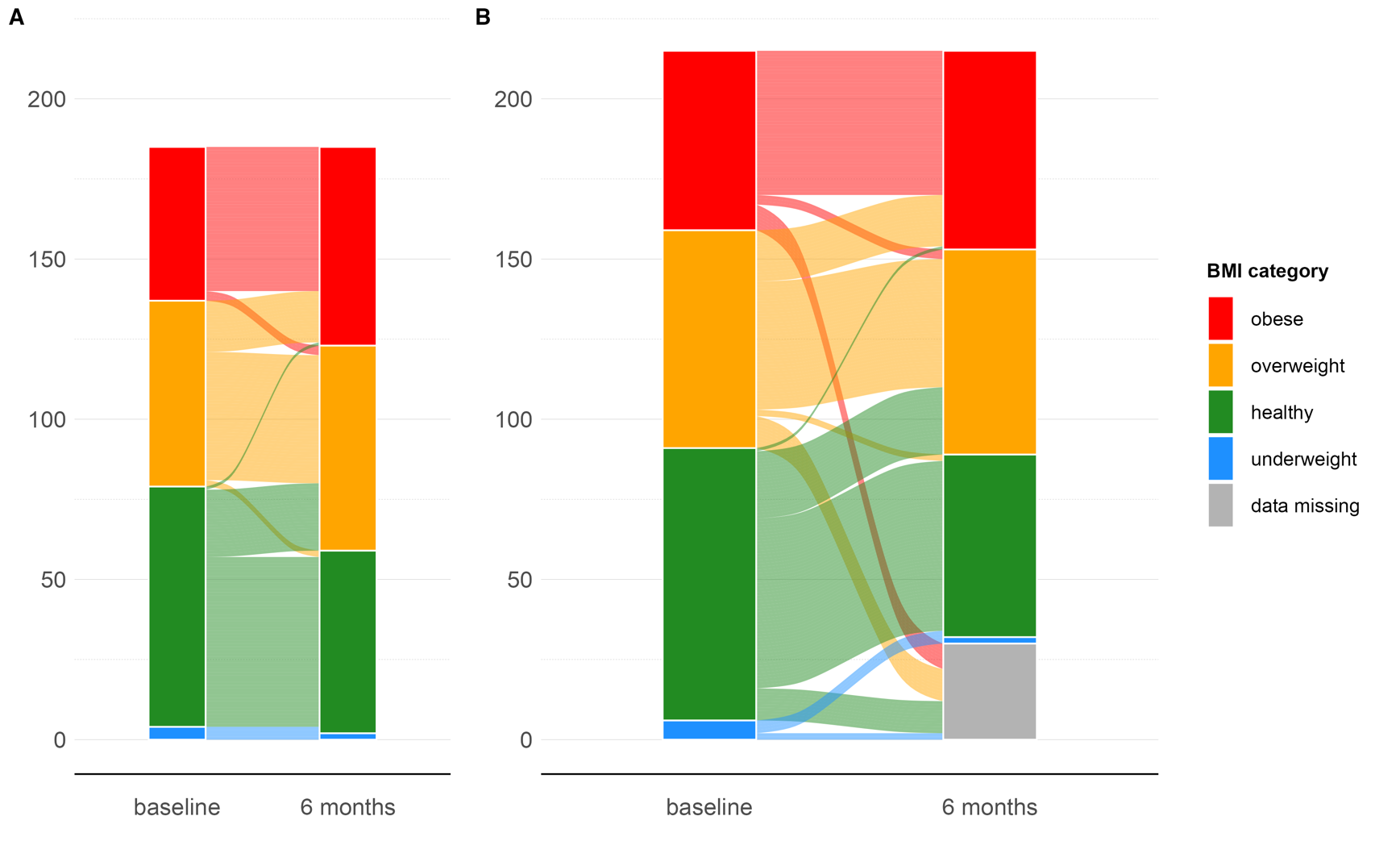
**

**Figure S2.** Percentage change in BMI at six months according to baseline BMI.


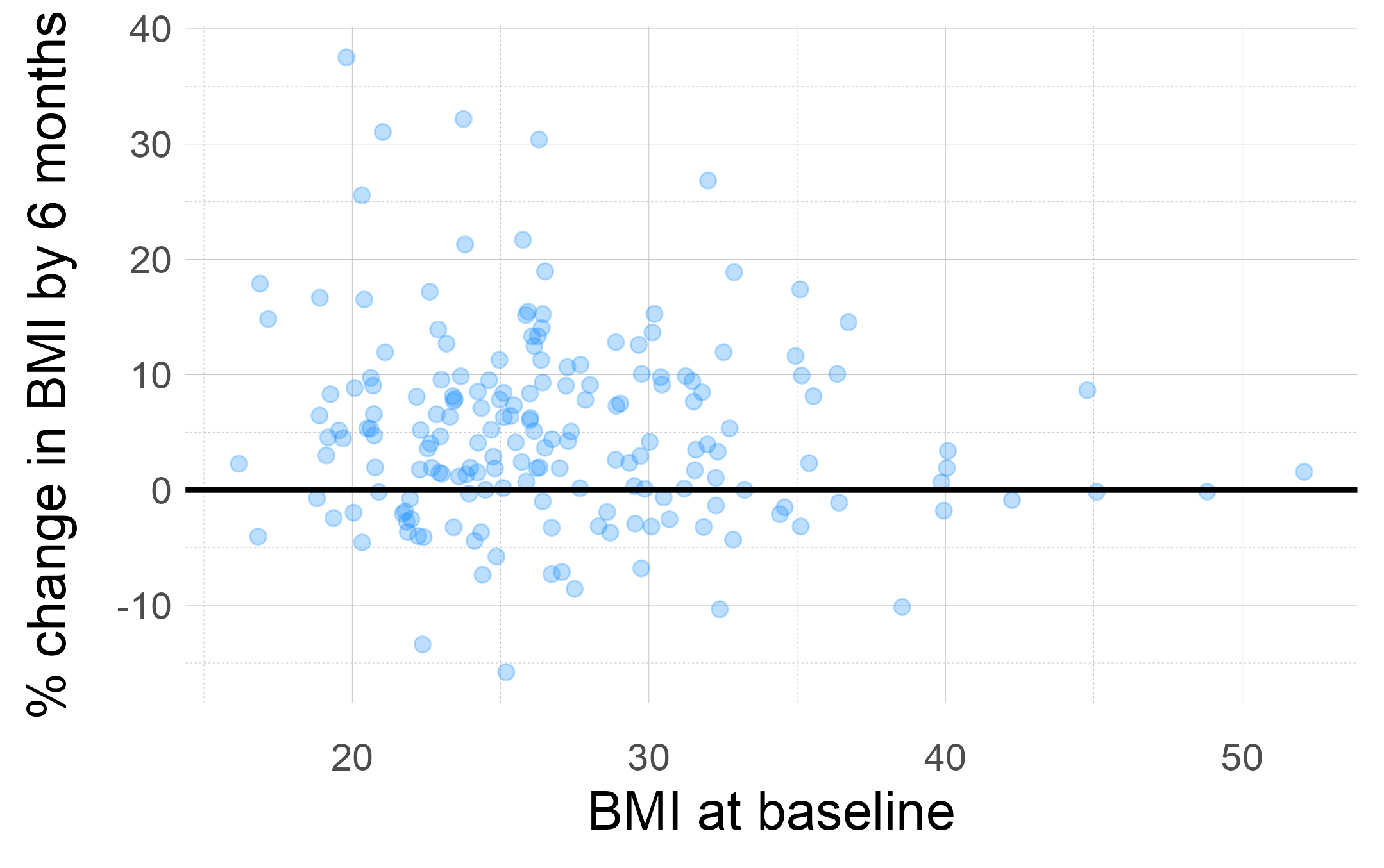


**Figure S3.** Percentage change in BMI at six months following AAV diagnosis grouped by baseline BMI category.

**
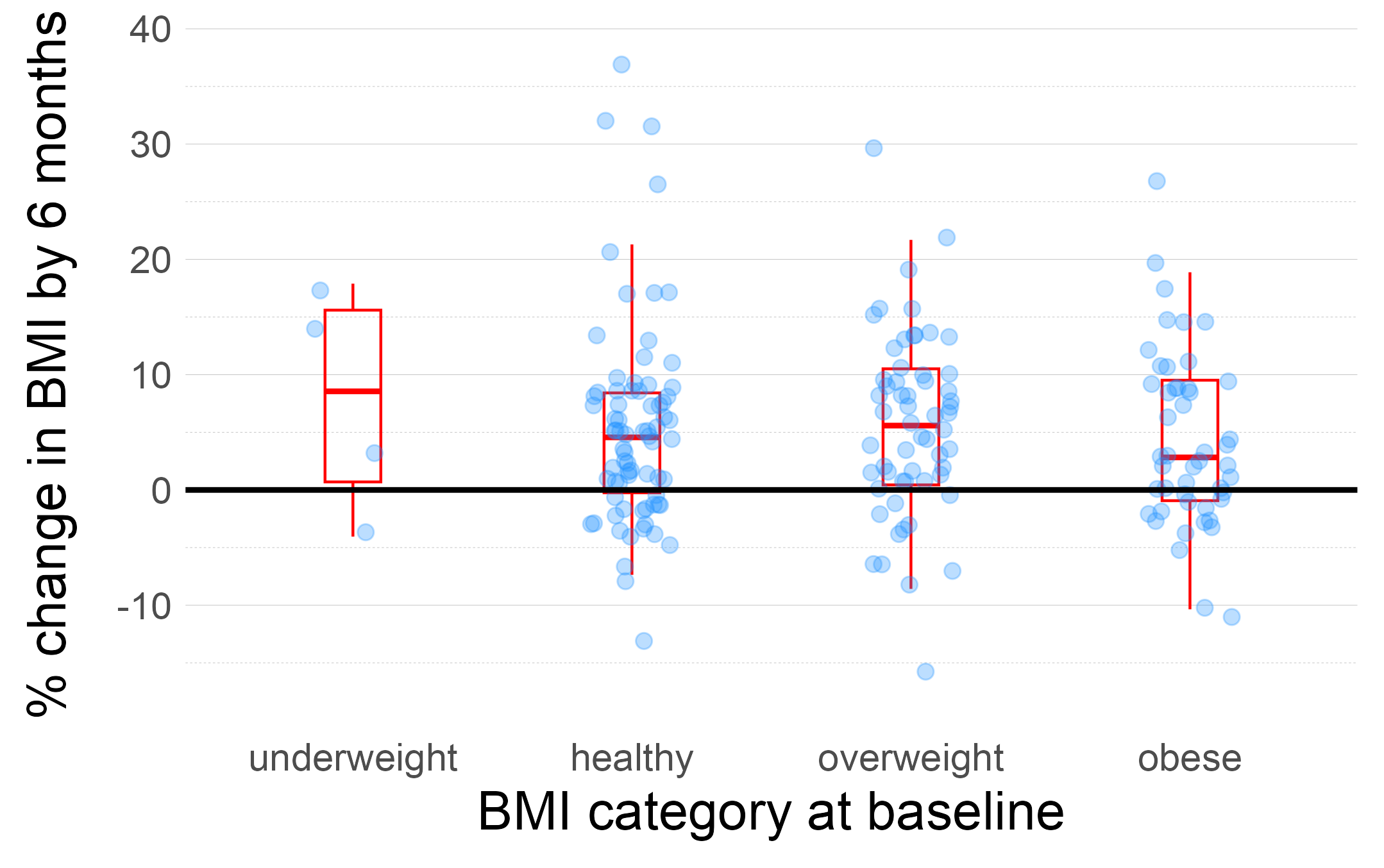
**

**Figure S4.** Kaplan-Meier plot on cumulative risk of relapse over time following initial presentation in obese and non-obese participants. Obese group in black, comparison group in red. Log-rank test P-value <0.900.

**
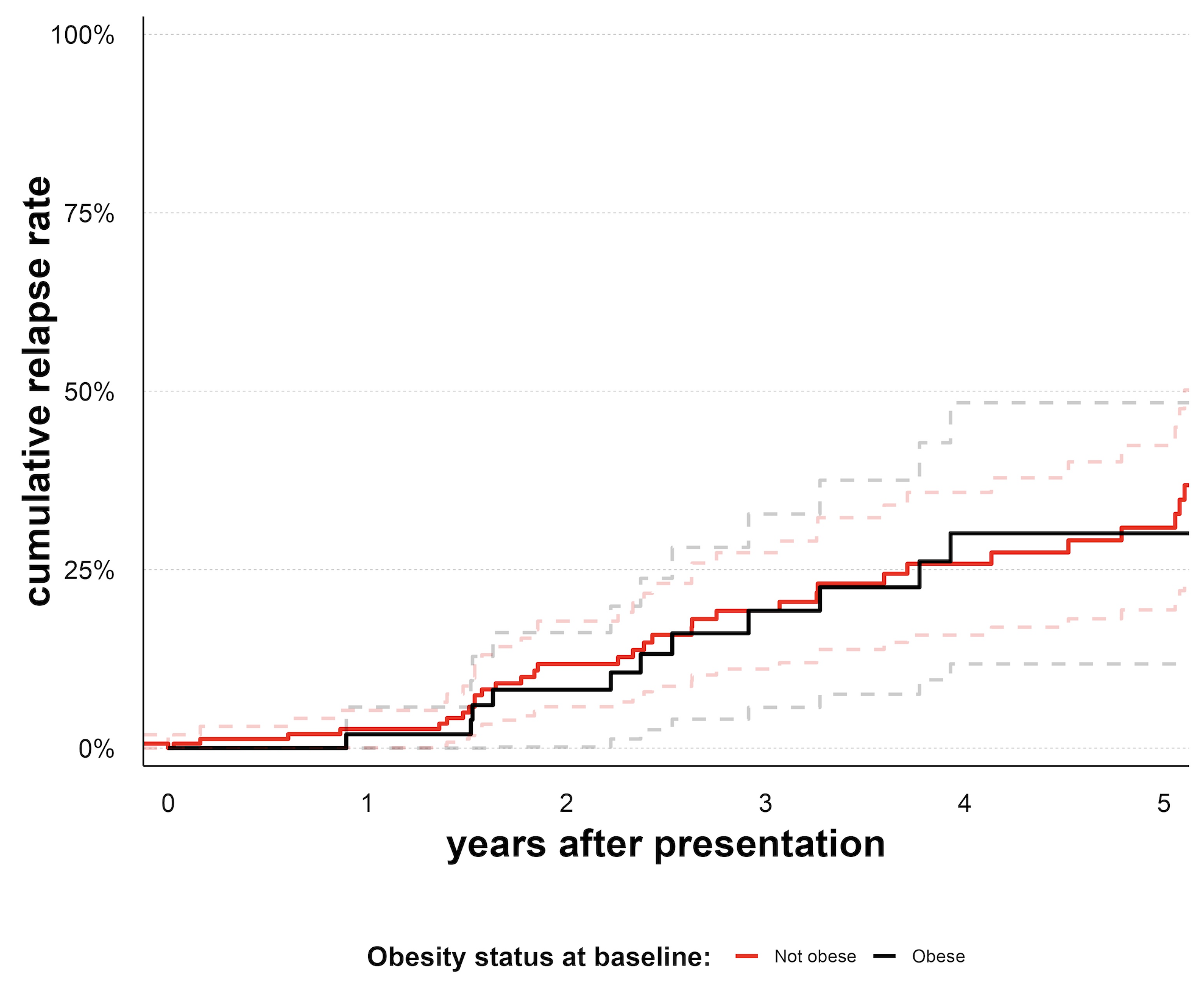
**

**Figure S5.** Percentage plot demonstrating changes in induction immunosuppression agents over time.

**
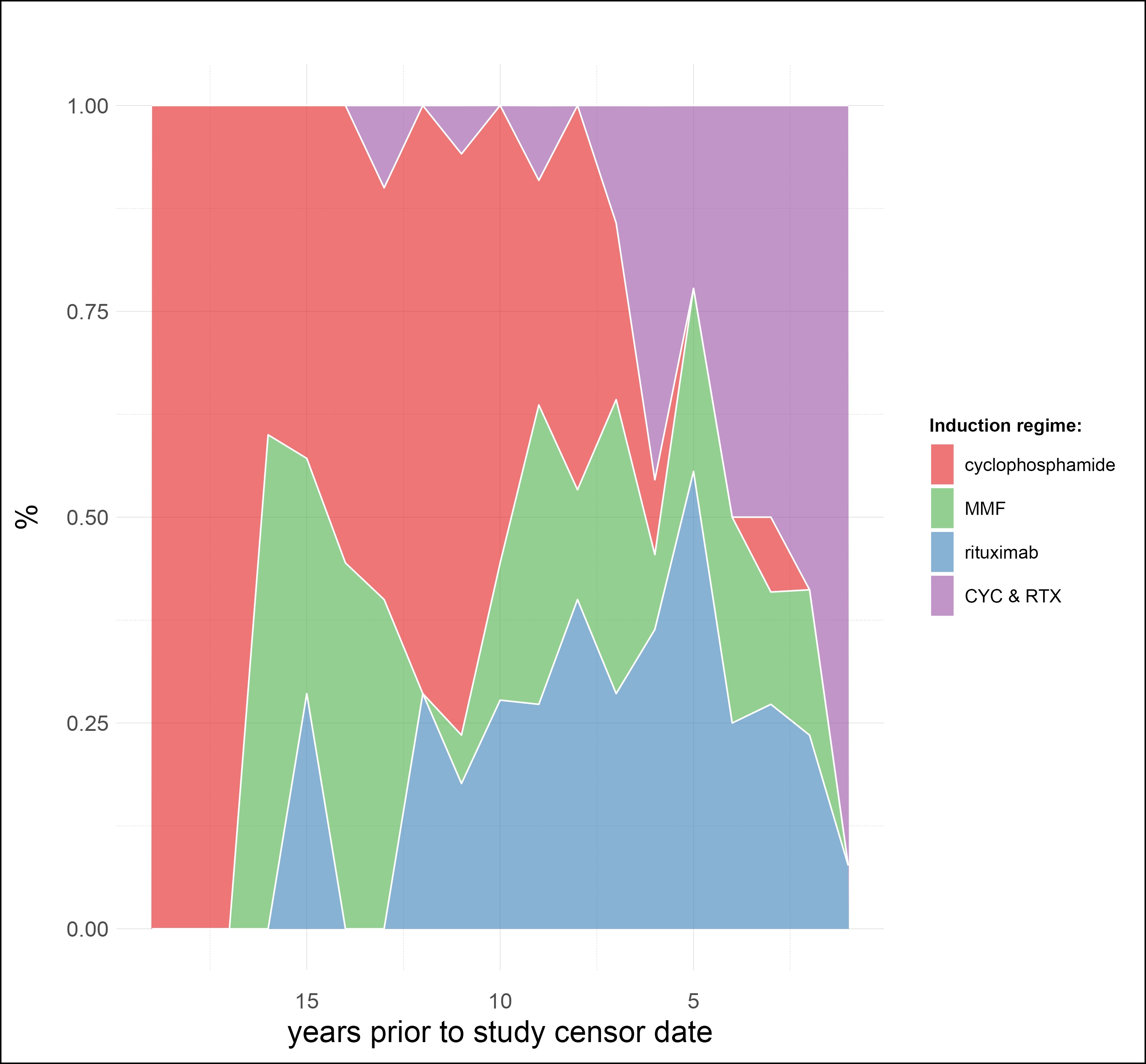
**

**Figure S6.** Prednisolone doses at presentation and three months, grouped by year of presentation to the vasculitis service.


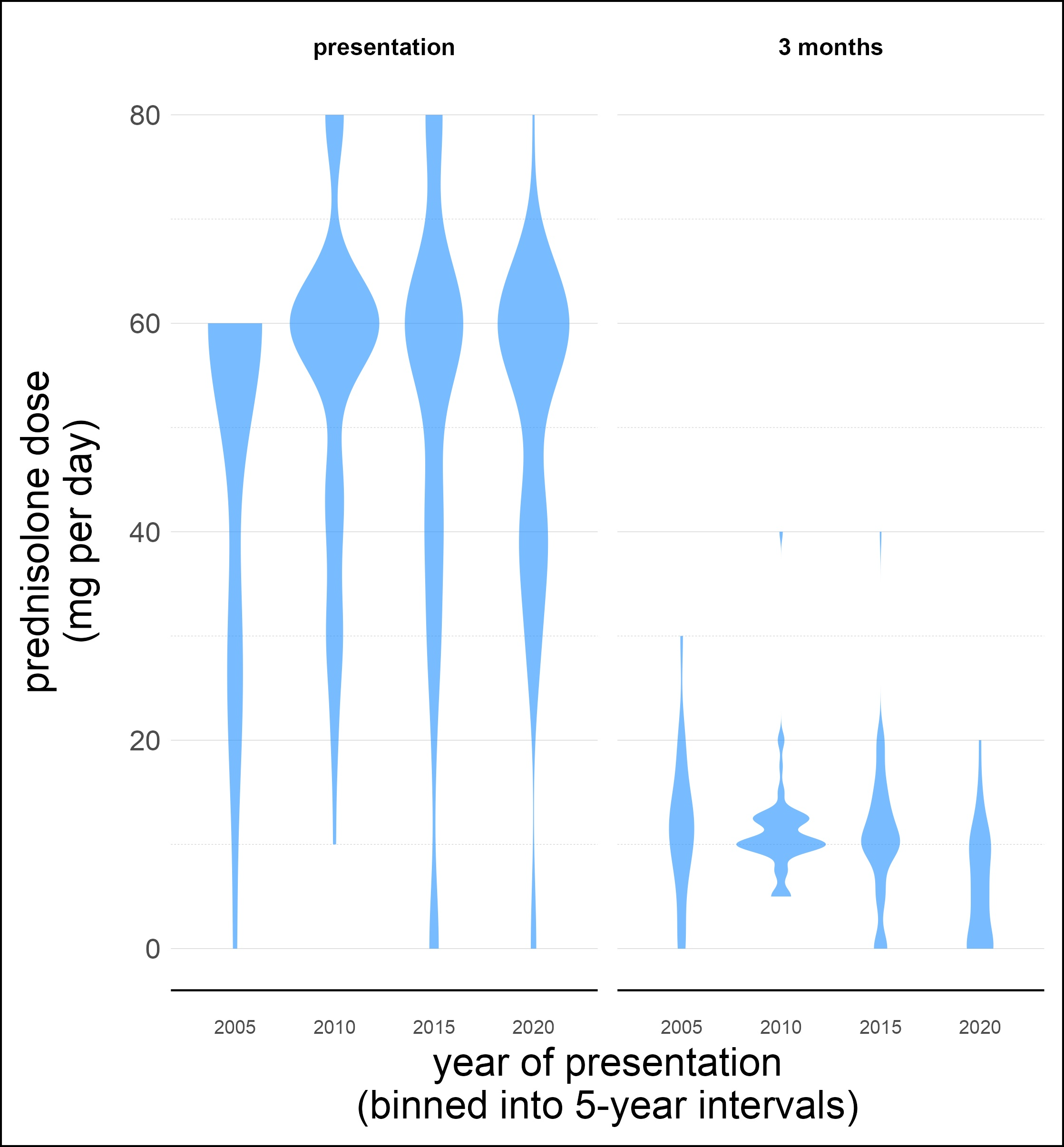

Supplement: rkaf088_Supplementary_Data [file rkaf088_supplementary_data.docx]
